# Measuring Social Sustainability in the Italian Agri-Food Sector: Proposed Key Performance Indicators

**DOI:** 10.3390/foods13172849

**Published:** 2024-09-08

**Authors:** Lucia Briamonte, Raffaella Pergamo, Chiara Salerno, Anna Uliano, Concetta Nazzaro

**Affiliations:** 1 CREA-PB, 00198 Rome, Italy; lucia.briamonte@crea.gov.it (L.B.); raffaella.pergamo@crea.gov.it (R.P.); chiara.salerno@crea.gov.it (C.S.); 2 Department of Law, Economics, Management and Quantitative Methods, University of Sannio, 82100 Benevento, Italy; cnazzaro@unisannio.it

**Keywords:** social sustainability, ESG, social sustainability indicators, Italy, agri-food sustainability

## Abstract

The social dimension of sustainability in the agri-food sector is gaining more and more attention from both scholars and policymakers. In Europe, among different countries, Italy stood out for the active role played in including social conditionality in the Common Agricultural Policy. Despite such interest, there is still confusion concerning the concept of social sustainability, and tools aimed at measuring the social performance of farms are still lacking. The current study aims to identify indicators to measure the social sustainability of farm practices in the Italian agri-food system. The methodology included an analysis of the most relevant literature, legislation, and guidelines to identify five macro-areas of interest, which served as the foundation for developing theoretical social sustainability key performance indicators. The results of this study provide useful insights for both practitioners and policymakers to develop strategies and policies focused on social sustainability.

## 1. Introduction

In recent years, social sustainability in the agri-food sector has gained increasing centrality in theoretical, institutional, and policy debates. In particular, the 2030 Agenda of the United Nations recognized social sustainability as a key pillar alongside economic and environmental sustainability [1]. Also, in the European Union, the 2021–2027 reform of the Common Agricultural Policy (CAP) introduced a social dimension pillar to promote decent work and ensure compliance with European labor law standards [2,3]. This has made social sustainability a requirement for agricultural funding [4,5,6]. Italy played a crucial role in advocating for social sustainability in Europe, leading to the inclusion of social conditionality in the CAP, ensuring the respect of labor and ethical standards in agriculture [7,8].

From a scientific perspective, although agriculture and agribusiness are frequently included in sustainability studies, the social dimension of agriculture remains relatively underrepresented [9]. Sustainable agriculture is typically examined in relation to environmental issues, such as the interaction between economic sustainability and natural resource conservation or the need for greater efficiency in resource use [10,11]. It is only in the last decade that the social aspect has become more integrated into the broader concept of sustainability. However, this progress has not led to a coherent understanding of what the social dimension should encompass [12,13,14,15]. Moreover, despite the interest in the definition of social performance indicators dating back to the 1960s, companies have only recently focused on indicators, allowing them to undertake a path of improving their social impact [16]. Social sustainability and related reporting in the last decade have aroused renewed attention from different actors belonging to the business, academic, and professional world, as well as public institutions. 

In virtue of the above, this study aims to develop a set of indicators to measure the level of social sustainability of farm practices in the agri-food system. In particular, the analysis focuses on the Italian context, as Italy has demonstrated an active role in social issues, both nationally and internationally.

Starting from a review of the literature on social sustainability in the agri-food sector, including national and international guidelines and measurement systems, this study intends to select and identify indicators to measure farms’ social sustainability performance based on validation through the literature selection criteria. 

The results of this study are intended to provide valuable insights to practitioners and policymakers to improve the performance of farms in terms of social sustainability, promoting an effective transition to more equitable and responsible agricultural and agri-food models.

This paper is organized as follows. The next section shows the background of this study, including a literature review and a review of the European and international institutional debate on social sustainability in the agri-food sector. Section 3 describes the methodology adopted, while the study results are presented and discussed in Section 4. This study’s conclusions, limitations, and implications are finally shown in Section 5.

## 2. Background of the Study

### 2.1. Social Sustainability in the Agri-Food Sector

The definition of sustainable development, which comes from the Brundtland Report in 1987, highlights the importance of equity within and between generations and recognizing that the Earth’s resources limit the ability to meet people’s needs [17]. Despite this definition focusing on people, several authors argue that the primary emphasis is on environmental sustainability [18,19]. In fact, the other dimensions of sustainability, such as the economic and the social, but particularly the latter, have often been put aside [13].

Today, research on social sustainability covers various fields, investigates various levels, and employs different conceptual approaches [20]. The topics encompass development studies, political studies [21,22], project development [23], and business and management studies [24,25,26,27], amongst many others.

Hart (1999) provides valuable examples to demonstrate the importance of integrating a society’s economic, social, and environmental aspects when developing indicators for a sustainable community [28]. She argues that economic indicators such as the Gross Domestic Product (GDP) only reflect the level of economic activity and overlook the impact of that activity on the social and environmental well-being of a community. As a result, GDP can increase while overall community health declines. Instead of using median income as an economic indicator, Hart suggests using the number of hours of paid employment at the average wage needed to meet basic needs. Additionally, the author proposes using the diversity and vitality of the local job market instead of the unemployment rate [17,28].

According to Amartya (1993), social sustainability has four dimensions: quality of life, equality, diversity, and social cohesion [29]. On the other hand, Eizenberg and Jabareen (2017) include four main concepts related to social sustainability: urban forms, equity (justice), eco-prosumption, and safety [13].

The Western Australia Council of Social Services (WACOSS) stated that social sustainability occurs when the formal and informal processes, systems, structures, and relationships actively support the capacity of current and future generations to create healthy and livable communities. Socially sustainable communities are equitable, diverse, connected, and democratic and provide a good quality of life [18].

In food-related studies, to explain the social sustainability concept, several scholars address participatory approaches [30] and social learning among farmers and rural communities [31,32,33] or consumers [34].

Sidhoum (2018) attempted to analyze the sustainability of social factors and their impact on the efficiency of agricultural production [35]. The author suggests a framework based on the state-contingent outputs to calculate shadow prices for social outputs. Janker and colleagues (2019) argue that the social sustainability of a system is determined by the degree to which needs and rights are satisfied [9]. Drawing upon Parsons’ social system of change, they combine the concepts of agricultural and social systems to identify the critical aspects of the social dimension of sustainability in agriculture [9,36]. They then incorporate the needs concept introduced by Maslow (1943) and the (human) rights approach by Gasper (2007) into their framework of social sustainability in agriculture [37,38]. These concepts are valuable as they establish the individual conditions necessary for overall societal well-being, thus bridging the gap between societal and individual levels of sustainability. Additionally, needs and rights align with the widely accepted definition of sustainable development [39].

Furthermore, in the agricultural sector, social sustainability is commonly evaluated throughout the food supply chain [40]. Some authors [41,42] examined social sustainability in specific supply chain stages. Other authors focus on analyzing social sustainability within a single stage of the supply chain and on one stakeholder without considering the existing relationships with other stakeholders [43,44]. Desiderio et al. (2022) reviewed the current state of the art in measuring social sustainability aspects using various tools and indicators throughout the food supply chain and among the actors involved at each stage [45]. The authors identified thirty-four social sustainability tools within five stages of the food supply chain: production, processing, wholesale, retail, and consumer. Such stages and stakeholders were adapted from the most recent guidelines for Social Life Cycle Assessment (SLCA) and include farmers, workers, consumers, and society.

According to the Sustainable Agriculture Research and Education Quality-of-Life Working Group (2021), social sustainability is defined as the degree to which social relationships foster fairness, justice, and a high standard of living [46]. Just as sustainable agriculture plays a role in maintaining long-term ecological health and economic viability, it also contributes to vibrant communities and regions and provides satisfying livelihoods for farmers, ranchers, and other individuals within the food system. The social term in the three dimensions of sustainability pertains to individuals’ different connections and interactions, both in person and within the broader food system. Social relationships can exist on different levels within sustainable agriculture, such as personal and household, farm or ranch, local community, agri-food network, and society. 

In summary, social sustainability within sustainable agriculture involves promoting fairness, justice, and a high standard of living through various levels of social relationships. This includes fostering personal, community, regional, and global connections and contributing to resilient and thriving agricultural communities and regions.

### 2.2. Social Sustainability, Policies, and Indicators: A Review of the European and International Institutional Debate

Social sustainability is particularly relevant in the agri-food sector, contributing to social justice, food security, the well-being of rural communities, and building a sustainable future for present and future generations [47].

The integration of social sustainability has been strengthened with the new CAP 2021–2027, which introduces the social dimension pillar, which emphasizes promoting decent work and ensuring compliance with European rules on workers’ rights [4,6].

Despite such recognition, the social sustainability of farms requires careful research and analysis to define indicators for its measurement. There is limited dissemination of organizations that have established specific tools to evaluate the social sustainability of farms. Notably, Fairtrade International is a non-profit organization that promotes fair trade globally. It manages the Fairtrade certification system and has developed specific guidelines and indicators for agricultural producers and farms, including the organization of producers, the protection of agricultural workers’ rights, good agricultural practices, and the prohibition of child and forced labor [48]. Another example is the Sustainable Agriculture Initiative Platform (SAI), which focuses on promoting social sustainability in agriculture. It has developed guidelines and standards addressing the health and safety of agricultural workers, workers’ rights, the involvement of local communities, and animal welfare [49]. The Global Social Compliance Programme (GSCP) is an international initiative that promotes social compliance in supply chains. It has created common guidelines and standards for social compliance, covering issues such as child labor, forced labor, worker safety and health, fair wages, and respect for fundamental human rights [50]. Finally, the Global Reporting Initiative (GRI) is an international organization that provides sustainability standards and is currently developing standards for agriculture, fisheries, and aquaculture, known as GRI 13, which have been in effect since 2024. These standards offer specific guidelines and indicators for assessing and communicating sustainability performance in these sectors [51,52].

Furthermore, some certifications aim to enhance the social sustainability of farms, though their use is still limited. In particular, Friend of the Earth, developed under FAO’s SAFA guidelines, focuses on ecosystem preservation and was introduced in 2016. It requires farms to follow specific guidelines on natural resource management, reduced chemical use, animal welfare, and social responsibility. GLOBALG.A.P. (Good Agricultural Practices), established in 1997, sets standards for responsible agricultural production, covering pesticide use, resource management, food safety, and worker health. Farms are certified through independent audits. Finally, Certification’s ESG Rating: 2020, originally for listed companies, is now applied to farms. It assesses environmental, social, and governance performance, aligning with ISO standards and UN SDGs, and provides a standardized framework for sustainability reporting.

Moreover, several projects have been developed to identify indicators and guidelines for measuring social sustainability. In particular, the Equalitas Project and the California Sustainable Winegrowing Program (SWP) are in the wine sector. The first one, the Equalitas Project, launched in 2015 in Italy, focuses on assessing the sustainability of wineries and currently has more than 250 certified companies.

The California Sustainable Winegrowing Program (SWP), launched in 2002 in the United States by the California Sustainable Winegrowing Alliance (CSWA), represents a collaboration between the Wine Institute and the Californian Association of Wine Grape Producers (CAWG). This program aims to improve sustainable practices in wineries and vineyards constantly.

Concerning European directives and regulations currently in force to promote farms’ social sustainability, there are Directive 2014/95/EU on the communication of non-financial information and information on diversity, Regulation (EC) no. 834/2007 on organic products, Regulation (EC) no. 1234/2007 on the labeling of agri-food products, Directive 2000/78/EC on equal treatment in employment and employment, and Regulation (EC) no. 1169/2011 on food information provided to consumers and rules on health and safety at work.

Furthermore, in June 2024, the EC launched the Agri Sustainability Compass, a new tool to measure agricultural activities’ sustainability performance [53]. Such a tool provides a set of indicators of economic, environmental, and social nature. As regards the latter, five indicators have been identified: antibiotics, age, gender, training, and poverty [53].

## 3. Methodology

This study aims to improve the understanding of the social dimension of sustainability in the agri-food sector. To this end, a set of indicators has been identified that, according to the peculiarities of the sector, allow for the standardization of the measurement of social performance and the identification of a model of synthetic reading of the most significant social aspects for the agricultural and agri-food sectors. In particular, the analysis was carried out through two phases: (a) identification of the priority dimensions to be explored (macro-areas) and (b) definition and summary representation of social sustainability indicators (KPIs). The first phase started with a literature analysis, with a particular focus on the leading practices of social reporting and sustainability, and standards of national and international bodies, as well as the main specific scientific publications on the topic. In particular, reference was made to the GRI Sustainability Report Guidelines (in particular GRI 13), reports and indicators of sustainable development (Italian Alliance for Sustainable Development—ASviS, United Nations), statistical databases (Eurostat, ISTAT, ILOstat, OECD), certifications and evaluation systems (CSR, SROI, SCBA, SLCA, ISO 26000, SA 8000), and EU directives and other policy tools.

**Figure 1:**
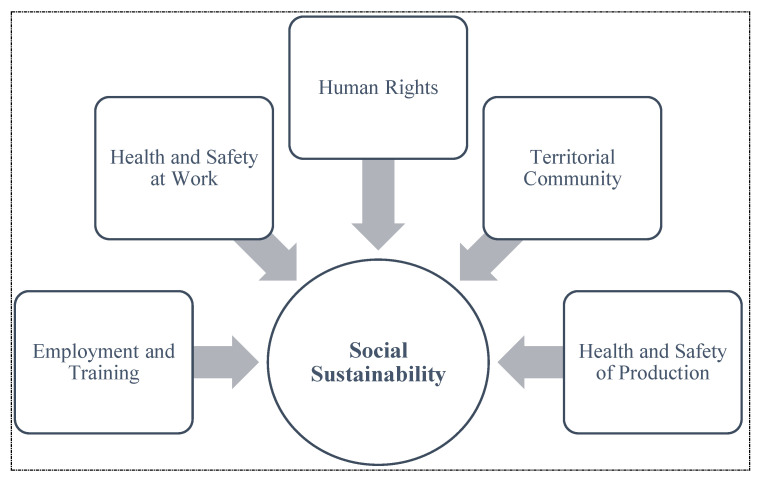
Social sustainability macro-areas.

This study aimed to capture the complexity of social issues in agriculture and its roots in the reference community, integrating the concept of needs introduced by Maslow (1943) and the (human) rights approach by Gasper (2007) [37,38]. Accordingly, the social sustainability of a system is determined by the extent to which needs and rights are met. Before defining the social sustainability indicators, five macro-areas were identified (Figure 1): employment and training (ET), health and safety at work (HSW), human rights (HR), territorial community (TC), and health and safety of production (HSP).

In this study, the measurement of social issues followed a subjective approach [54]. The latter is used in the GBS Principles of Social Reporting and is based on the categories of reference stakeholders. Such an approach is consistent with social responsibility as a responsible management of stakeholder relations [55]. Each macro area represents a stakeholder, a part of the social performance measurement system in the agri-food sector aimed at framing the relationship between the farm and the specific stakeholder. The macro-areas help to identify and describe the relevant peculiarities of the specific category of stakeholders and, therefore, to identify more precisely the individual indicators.

The selection of indicators was guided by the concept introduced by Gallopin (1996) [56], which offers a broad and inclusive definition. Gallopin asserts that indicators are not values themselves but variables, serving as an operational representation of an attribute (such as a quality, characteristic, or property) of a system. This study began by analyzing existing indicators, then adapted them to the sector and introduced new, flexible, and scientifically grounded indicators that had not been previously addressed [16]. Ultimately, the indicators were chosen based on the following criteria [57] (Table 1): ease and understanding, significance, inclusiveness, manageability and comparability, controllability, continuity, and efficiency.

## 4. Results and Discussion

The qualitative analysis of the concept and tools for assessing farms’ social sustainability reveals significant variability in the social dimension, particularly in terms of scope and standards applied [9,21]. Although specific tools for assessing social sustainability are not widely used, many best practices exist that can help develop a comprehensive set of indicators [58,59]. The KPIs identified for each macro area will be discussed in the following sections. 

### 4.1. Employment and Training (ET) Macro-Area

The ET macro-area has the following purposes: promotion of decent work; employment growth and stabilization of income; education and training of employees, encouraging the entry and retention of young people and new entrepreneurs; and support for reconciliation between work and family.

Decent work is a multidimensional concept that integrates universal human rights, individual needs, and social justice, encompassing normative, contractual, relational, and political aspects [60,61]. To select appropriate indicators, attention was given to areas such as combating illegal hiring, employment growth, income stabilization, education and training, youth retention, internal communication, work environment quality, and support for work–family balance [62]. Five indicators have been identified (Table 2) that contribute to our framework of social sustainability in agriculture to integrate Maslow’s needs (1943) and the approach to human rights (Gasper, 2007) [37,38]. For this reason, the dimension of work becomes the keystone, contributing to ensuring social and individual aspects of well-being and sustainability [61]. This method is widely used by major international bodies, such as the United Nations and OECD [63].

The first two indicators, employment policies and development of human resources (ET1) and employees’ training and professional growth (ET2), are cross-sectional indicators that can adapt to different sectors and derive from indicators already present in the GRI guidelines and the ILOStat database. Such indicators have both qualitative and quantitative measurements.

For the development of human resources, particular attention was paid to the foreign labor force (specific indicator training for foreign employees—ET3). The enhancement of human resources, in all its forms, is one of the individual preconditions for overall social well-being and thus allows the connection of the level of social sustainability with the individual, in line with the more accepted definition of sustainable development [39]. In particular, a functional and efficient farm requires more and more new skills and knowledge, a design capacity, the introduction of new activities, a reorganization of company resources in the function of new technological models and relationships with the market, and the establishment of new relationships between company resources and territorial resources [64]. The strengthening and valorization of the human capital and the territorial capital become essential elements to operate on the market and grasp the complex interrelations between the various functions found both at a single unit of production level and the territorial one.

The following three indicators are specific indicators identified according to the peculiarities of the Italian agri-food sector. In particular, reference was made to the seasonality of production and the possibility of the company using a farm labor requirement plan (ET4) for a valuable forecast of such needs. This requires a strong capacity for innovation from both farms and in the context of policies and their management [65]. 

Finally, for a better guarantee of compliance with labor standards, social legislation, income tax, and value-added tax, reference is also made to membership in the network of quality agricultural work (ET5).

### 4.2. Health and Safety at Work (HSW) Macro-Area

The HSW macro-area includes four indicators designed to measure how deeply safety is integrated as a core value in both work and private life. Given the obligations laid down by current legislation, the indicators (Table 3) aim to characterize all business practices aimed at promoting and protecting the health and safety of employees through quantitative and qualitative measurement.

The macro-area has been structured taking into account not only the practices undertaken and explicitly related to the production process, such as the scheduled maintenance of machines and equipment, but also the actions aimed at employees, including, for example, vouchers for medical examinations for work-related risk diseases or, more generally, insurance policies; the number and costs incurred annually in safety equipment and devices; the prevention of accidents at work; and safety of workplaces, in addition to those resulting from current legislation.

In particular, the first occupational health and safety policies and practices (HSW1) indicator focuses on the practices adopted in favor of occupational safety, with a particular focus on the procedures for planning interventions and the extraordinary maintenance of machinery and equipment. The indicator is fully reflected in the document drawn up by the Italian Institute for Insurance against Accidents at Work (INAIL), “Maintenance for safety at work and safety in maintenance”, as well as with the INAIL facilities for reducing the average tariff rate for companies that have carried out measures to improve health and safety in the workplace, in addition to those provided for by the relevant legislation (interministerial decree of 27 February 2019, Minister of Labour and Social Policy). 

The second indicator, training and information initiatives on health and safety at work (HSW2), aims to measure the actions undertaken to encourage the empowerment of the actors that contribute to constructing a solid information culture as a tool for subjective prevention going beyond the mere obligation regulatory.

The employee vouchers and insurance policies (HSW3) indicator focuses on those voluntary initiatives that provide vouchers for medical examinations to employees as part of their welfare programs or company policies. These benefits may cover expenses for periodic medical examinations, diagnostic examinations, or other preventive medical services to promote employees’ health and well-being and identify any health problems promptly. 

The specific consulting (HSW4) indicator, in line with the innovations introduced by the Regulation (EU) 2021/2115, focuses on the activation of specific counseling services as a tool that can contribute to the strengthening of prevention actions by the employer; the indicator has been developed in line with the Guidelines for Sustainability Reporting on Working Practices and Adequate Working Conditions Indicators [66].

### 4.3. Human Rights (HR) Macro-Area

This macro-area aims to overcome diversity and promote equity, inclusion, and equality through companies’ due diligence. As McKenzie (2004) argued, socially sustainable communities are fair, diverse, connected, and democratic and provide a good quality of life [18]. The concept of equity and equality also reflects the four dimensions of social sustainability identified by Amartya (1993): quality of life, equality, diversity, and social cohesion [29]. There is a growing interest in diversity, equal opportunities, and inclusion [67,68]. The company, in fact, is in a context from which it cannot prescind and looks for a kind of legitimization of its activity. Creating economic and social well-being thus becomes the minimum requirement for a positive relationship between the enterprise and its reference context. Welfare can be created in various ways, as shown by the indicators in Table 4.

More specifically, for human rights, reference is made in this context to the second pillar, “Corporate responsibility to respect human rights”, of the Guiding Principles on Business and Human Rights approved in 2011 by the UN Human Rights Council. The Guiding Principles are based on three pillars: (a) the state duty to protect against human rights abuses by third parties, including business; (b) the corporate responsibility to respect human rights; and (c) greater access by victims to an effective remedy, both judicial and non-judicial [69].

In light of the above, the UN Human Rights Council and the European Union asked Member States to develop appropriate action plans to address and manage the implementation of the principles at the national level. 

From an operational point of view, the impact of business activities on human rights can affect many actors (workers, migrants, and children), take different forms (discrimination, exploitation, pollution, etc.), and register in different economic contexts (agriculture, textiles, finance, energy resources, etc.), potentially revealing itself to be much wider, possibly including every aspect of contemporary society [70]. 

In this direction, human rights indicators relate to concrete information on the status of an object, event, activity, or outcome that can be used to assess and monitor the promotion and protection of human rights. There are many indicators relating to respect for human rights, but they are often not of a quantitative nature; they could also be classified as indicators based on facts and judgment, corresponding to the category of objective and subjective indicators in the literature on statistics and development indicators [71]

Given the above, five indicators (two cross-sectional and three specific) have been identified in this macro-area. In particular, the cross-sectional indicators relate to including human rights clauses in contracts and agreements with third parties and throughout the supply chain (HR1) and the initiatives launched favoring non-discrimination (HR2).

As regards specific indicators, the focus has been on adequate remuneration (HR3) concerning the sector’s wage levels in order to ensure fair pay, positive action to protect women and children (HR4), and, finally, social inclusion actions (HR5) through community involvement (i.e., support for transport, housing, and schools).

### 4.4. Territorial Community (TC) Macro-Area

The TC macro-area aims to highlight the importance of the contribution of the farm to its territorial community, taking into account the fundamental role of the surrounding environment in competitive growth and increased overall well-being.

Farms’ sustainability involves a complex process where monetary value is interconnected with personal, family, and environmental factors, as well as economic, social, cultural, and institutional contexts, all of which contribute to the welfare process. Based on this understanding, the indicators identified (Table 5) assess the transition from merely having resources to the ability of individuals to achieve goals and improve quality of life and well-being. These indicators focus on shifting attention from market goods to relational goods.

Accordingly, two specific indicators have been identified: liberality initiatives (TC1) and territorial networking (TC2). Such indicators highlight the aggregation capacity of farms and the level of networking reached, social activities of inclusion and enhancement, transparency and dissemination of useful information to consumers, and the voluntary initiatives undertaken. Such elements are functional to highlight the link between farms and their territorial communities [72]. Moreover, the territory coordinates its resources and economic activities in a perspective of harmonization with the cultural and natural traditions of the place itself [73]. The territory is the place where the farm finds the reasons for its being and its becoming, drawing on the available social capital. Therefore, there is the creation of local economies finding their competitive advantage in the interdependence and complementarity of their formulas. Such development does not always arise spontaneously but often needs stimuli and coordination that can come from local institutions, which should identify the consistent trajectories of growth of the territories considering their vocations and potential to achieve balance [74]. For the agricultural sector, the “territory–product” relationship makes the typicality and uniqueness of the offer and makes the production of a competitive territory, as it cannot be replicated. Social value is achieved when uniqueness is combined with notoriety, thus elevating the territory to a fundamental element for the attractiveness of its places and contents. The territory can thus become a bulwark placed to defend the specificities of production, traditions, and culture of places, proposing a different offer from the logic of globalization. Here, the cross-sectional indicators of transparency of information (TC3) and social activities and local projects (TC4) are identified.

According to Dalla Chiara et al. (2014), the relationship between the territory and well-being results from numerous components and is not dependent on income alone [75]. In particular, the levels of families’ well-being tend to decrease in a limited way, passing from urban to fully rural areas. The measure of well-being is, therefore, the result of a multidimensional evaluation process, no longer linked to a single indicator, as is generally the case with the measurement of per capita GDP, as well as the quality of life depends on people’s conditions, such as health status, access to education, personal development, sufficient environmental conditions and investment in social capital. The cross-sectional indicator networking (CT5) falls under this last consideration.

### 4.5. Health and Safety of Production (HSP) Macro-Area

This macro-area aims to highlight how the theme of public health and consumer protection affects the entire agri-food chain in an integrated manner.

The European Union regulates food safety policy with two articles—168 and 169—in the EU Treaty of Operation, which include aspects of safety of primary production, hygiene conditions in food processing, packaging, labeling, and official controls on compliance with food safety. More generally, food security and food safety are factors characterizing consumers’ choices, but they are also an indirect measure of the changes in the economic–productive, environmental, and ethical social assets of a productive context. Sustainability must affect the entire production chain, from upstream to downstream, and from this perspective, indicators have emerged to measure the attention of farms to certain aspects of farm life with the use of ethical certifications, the short supply chain, the use of digital instrumentation, the number of communication actions aimed at raising consumer awareness, and even measuring the importance of the area devoted to sustainable production systems.

Four specific indicators and one cross-sectional indicator have been identified, which are useful to highlight the importance of health aspects, particularly in Italian food production, and the proactive role of consumers (Table 6).

One of the main objectives of the Italian agri-food policy is the protection of production quality, as Italy stands out in Europe for the most significant number of registered trademark products. The qualitative components also include ethical and social factors, such as sustainable and fair-trade production methods, ecosystem protection, compliance with animal welfare standards, and respect for human rights and workers in the company and along the supply chain. The numerous certifications, including mandatory ones such as the International Organization for Standardization (ISO) (UNI EN ISO 9001:2015—quality management systems; requirements the UNI EN ISO 14001:2015—environmental management systems; requirements and guidance for use the UNI EN ISO 22000:2018—management systems for food safety; requirements for any organization in the food chain UNI EN ISO 22005:2008—traceability in agri-food chains; and general principles and basic requirements for system design and implementation), also go in this direction.

One of the certifications that consider the respect of human rights and employees is SA 8000, which encompasses nine social requirements aimed at increasing the competitive capacity of companies that voluntarily provide guarantees of the ethical nature of the supply chain production and the production cycle itself. Food production substantially impacts the individual and social spheres [76]. Given that the supply of raw materials and the processing processes are very complex, it is increasingly important to ensure a traceability system that identifies the entire life cycle of a product. The use of a QR code, blockchain, and other digital tracking systems is the last frontier to ensure transparency and traceability of data on the origin, quality, and status of food, maximizing the effectiveness and efficiency of business processes, differentiating from local and international competitors to ensure product excellence [77]. Very concrete social benefits are derived from short food supply chains, which are considered an accurate model for increasing transparency, trust, equity, and growth. Such a supply chain also strengthens the networks of communities, the contractual power of the actors, cooperation and solidarity, consumer awareness, gender equality and social inclusion, and transparency of information [40,78]. The identity of the agri-food system is found, then, not only in the production phase but also in that of market access and consumer awareness, which looks at all the product’s characteristics, not only nutritional but also territorial and human [79]. Finally, as already pointed out, the growing attention to sustainable cultivation methods, also recommended by the Farm-to-Fork Strategy, implies a review of the functioning of agricultural systems, including ecosystem protection and resilience to climate change. Access to safe food is a requirement overcome by a vision of food sovereignty that implies a national food policy that protects, in particular, small producers and key sectors from market speculation [80].

## 5. Final Remarks

Over the last two decades, the social aspect of sustainability has become increasingly crucial regarding policies and business strategies. The first purpose of this study was to address such an issue, highlighting the most significant aspects of the peculiarities of the agri-food system. Assessing social sustainability in the agri-food system aspiring to be rigorous is problematic due to the difficulty of identifying specific indicators. There are, in fact, several aspects of social sustainability that need to be considered for the agri-food sector and that can concretely respond to the reporting needs of enterprises.

Therefore, the focus of this study has been on identifying indicators through the analysis of the legislation, guidelines, and scientific literature on the topic, also providing new ones that take into account the peculiarities of the sector. 

The results of this study provide a contribution to the Italian agri-food sector by offering a set of KPIs to measure social sustainability. The identification of 24 distinct KPIs, organized into macro-areas of interest, such as employment and training, health and safety at work, human rights, territorial community, and production health and safety, allows farms to assess and improve their practices in line with social expectations and European policy directives. 

Some practical and managerial implications can be outlined from this study’s results. In fact, the KPIs could represent a practical tool for the continuous monitoring of social performance for Italian farms, helping to identify areas for improvement and develop strategies to enhance working conditions and community sustainability. The adoption of these indicators can also support farms in complying with Italian and European regulations on social sustainability, thereby improving their ability to create value not only economically but also socially, in addition to market reputation. Additionally, such KPIs can be useful for policymakers to guide the definition of sectoral policies that promote social sustainability, facilitating alignment between business goals and sustainable development objectives at regional and national levels.

While this study provides useful insights for measuring social sustainability in the agri-food sector, it has some limitations that need to be considered. Firstly, the identified indicators are theoretical, mainly derived from a review of the existing literature, regulations and international institutional debate, without empirical validation through application to a sample of companies. This implies that the effectiveness and practical applicability of these KPIs may vary when implemented in real business contexts. Secondly, since this study focuses on the Italian context, the conclusions may not be fully generalizable to other countries with different regulations and social conditions. 

Future studies could explore the validity and reliability of these KPIs by empirically applying them to a sample of Italian farms, taking into account structural and sectoral differences, to develop a deeper and more applicable understanding of social sustainability in the agri-food sector.

## Figures and Tables

**Table 1:** KPI selection criteria.

Ease and Understanding	Indicators must be selected primarily based on their comprehensibility and usability; if an indicator is not immediately understandable (complex or inconsistent), its usefulness is limited, both as an internal governance tool and as a means of communication to the outside.
Significance	Indicators should be significant and balanced to support decision-making by identifying spaces and opportunities for improvement.
Inclusiveness	The selected KPIs should be able to cover all major aspects and significant impacts.
Manageability and Comparability	The performance developments identified by the indicators should be assessed by ensuring the comparability and replicability of the results. If the indicators are developed based on recognized standards, they also offer the possibility of a continuous benchmark concerning the competitive arena.
Controllability	The controllability of an indicator depends on the entrepreneurial capacity to influence that indicator by its actions. This allows us to clearly account for the progress achieved by stakeholders.
Continuity	An indicator must be continuously updated and monitored to allow effective tracking of changes in performance.
Efficiency	Indicators requiring excessively expensive data collection or for which it is not technically possible to collect data are redundant and negatively impact performance by the resources used to compose them.

**Table 2:** Employment and training (ET).

KPIs	Qualitative Measurement	Quantitative Measurement	Reference
ET1: Employment policies and development of human resources	Describe the employment policies and activities undertaken to improve the working conditions of the employees (including family employees). Provide 1. the total number of employees divided by types—(a) type of contract; (b) gender, age, disability—and 2. age, gender, and education of the entrepreneur.	Total number of employees and rates of recruitment and staff turnover by age and gender in the last two years. Number and cost of actions taken in favor of human resources. Average hourly earnings of employees.	LA 2—Guidelines for Sustainability Reporting 2000–2011 Global Reporting Initiative (GRI) (indicators 1 and 2) 8.5.1—ILOStat database (indicator 3)
ET2: Employees’ training and professional growth	Describe any employee training activities carried out.	Number of employees who have attended company-sponsored training courses out of total employees in the last two years.	LA 10—GRI
	Describe the training results achieved in the field of employees’ professional growth.	Number of hours of training provided to employees in the last two years.	LA 10—GRI
ET3: Training for foreign employees	Describe the type of training activities activated by the company, particularly the Italian language training for foreign workers, and the possible attention to the aspects related to prevention and safety and final tests to assess their linguistic competence. Other specific training programs for foreign workers.	The number of courses and hours of specific training for foreign employees activated in the last 2 years.	Introduced by the authors (based on INAIL OT-24 SSL and HR 3—GRI)
ET4: Farm labor requirement plan	Use of a planning and scheduling tool for company labor requirements.		Introduced by the authors
ET5: Network of quality agricultural work	Registration in the network of quality agricultural work.		Introduced by the authors

**Table 3:** Health and safety at work (HSW).

KPIs	Qualitative Measurement	Quantitative Measurement	Reference
HSW1: Occupational health and safety policies and practices	Describe the actions that go beyond those provided in the existing legislation on the health and safety of the worker (e.g., equipment and devices for safety, prevention of accidents at work, and safety of the workplace).		INAIL OT-24 SSL
	Describe whether the company uses a specialized firm for the scheduled maintenance of equipment, machinery, or installations.	Number of maintenance and overhauls of equipment in use (beyond those resulting from applying the legislation) in the last 2 years.	
	Describe any occupational health and safety (OH & S) management systems.		
HSW2: Training and information initiatives on health and safety at work	Describe any training and information initiatives on health and accident prevention in addition to those provided for by law, e.g., BBS (Behavior-Based Safety).	Number of training and information initiatives in the last 2 years. Number of risk assessment and liability actions for companies employing seasonal workers for less than 50 days/year.	LA 10—GRI LA 8—GRI
HSW3: Employee vouchers and insurance policies	Description of the types of vouchers or benefits for medical examinations on diseases at risk related to the activity and insurance policies.	Costs incurred annually for the planned initiatives. Number of adhesions /total employees. Number of insurance policies for employees.	LA3—GRI
HSW4: Specific consulting	Provision of specific counseling services on health and safety at work, employment conditions, and social assistance.	Number of counseling services activated in the last two years.	LA 8—GRI

**Table 4:** Human rights (HR).

KPIs	Qualitative Measurement	Quantitative Measurement	Reference
HR1: Human rights clauses	Describe the types of human rights clauses included in contracts and agreements with third parties (e.g., inclusion of agreements on the prohibition of exploitation of women and child labor).	Percentage and total number of significant investment agreements, including human rights clauses.	HR 1—GRI
HR2: Non-discrimination initiatives	Describe the presence of protected categories and diversity situations.	Number of female employees/total employees.	HR 4—GRI
		Number of immigrant employees/total employees.	
	Describe the voluntary actions undertaken in favor of non-discrimination.	Number of protected or weak categories employees/total employees.	
		Number of non-discrimination actions taken.	
HR3: Adequate remuneration		The difference between wage and salary levels in the reference sector.	Introduced by the authors
HR4: Female and child labor	Farm’s policies to protect women and child labor.	Number of initiatives to protect women, children, and minorities.	Introduced by the authors
HR5: Social inclusion	Types of actions in favor of the social inclusion of workers (e.g., number of dedicated means of transport, access to schools, language courses, dedicated accommodation).	Number of actions in favor of the social inclusion of workers (e.g., number of dedicated means of transport, access to schools, language courses, dedicated accommodation).	Introduced by the authors

**Table 5:** Territorial community (TC).

KPIs	Qualitative Measurement	Quantitative Measurement	Reference
TC1: Liberality initiatives	Description of the social, cultural, charitable, and recreational activities in which the farm is involved.	Number of social, cultural, charitable, and recreational initiatives in the last two years.	Italian Revenue Agency
TC2: Relations with institutions, bodies, and organizations operating in the territory, territorial networking	Description of the farms’ relations with the public administration, institutions, territorial community, local authorities, etc.	Number of awareness and promotion initiatives organized over the last two years.	SO 5—GRI SO 6—GRI
TC3: Transparency of product information and promotion of healthy lifestyles	Disclosure of information on product and process characteristics, in addition to mandatory and optional information required by law. Dissemination of information on the nature, scope, and effectiveness of any program and practice promoting access to healthy lifestyles.	Amount of awareness and promotion initiatives in the last two years.	SO1—GRI
TC4: Social activities and local projects	Participation in voluntary rural development projects and/or initiatives to integrate agricultural products, crafts, and tourism. Restoration and territorial redevelopment. Guided tours in rural areas or forests or involving local partners from other sectors. Social inclusion initiatives for the benefit of the community.	Number of initiatives and/or projects in the last two years.	SO 9—GRI SO 10—GRI
TC5: Networking	Adherence to farm networking, which increases the value of the agricultural economy and territory.	Number of network activities.	Introduced by the authors

**Table 6:** Health and safety of production (HSP).

KPIs	Qualitative Measurement	Quantitative Measurement	Reference
HSP1: Ethical certifications		Number of ethical certifications	Accredia database SA 8000
HSP2: Digital equipment	Digital instrumentation used to increase production security (precision agriculture, blockchain, etc.).	QR code activation.	Italian Trade Agency (ITA)—Digital drawer—Track-it
HSP3: Short food supply chain	Input supply systems through short supply chains and purchasing groups, as well as supply chain control.	Seasonal products, number of ethical purchasing groups, marketplace, and e-shop managed by producers.	Eurobarometer 2019 Italian Institute of Services for the Agricultural Food Market (ISMEA)
HSP4: Consumer information	Communication actions to raise awareness and inform consumers about the health and safety of production.	Customer satisfaction practices	Italian Council for Agricultural Research and Analysis of Agricultural Economics (CREA)—Regional Hygiene and Nutrition Services
HSP5: Percentage of agricultural area devoted to productive and sustainable agriculture	Sustainable production methods.	The ratio between the area devoted to sustainable and productive agriculture (e.g., organic) and the total agricultural area (%).	Italian National Institute of Statistics (ISTAT) Italian information system on organic farming (SINAB) SDG Indicator 2.4.1

## Data Availability

Dataset available on request from the authors.

## Glossary

CAP	Common Agricultural Policy
GRI	Global Reporting Initiative
KPI	key performance indicator
ESG	environmental, social, and governance
ET	employment and training
HSW	health and safety at work
HR	human rights
TC	territorial community
HSP	health and safety of production
